# Influence of Periodontal Status and Prosthetic Treatment on Survival and Success Rates in Implant Therapy: A 5-Year Retrospective Follow-Up Study

**DOI:** 10.3390/jcm12134275

**Published:** 2023-06-26

**Authors:** Darius Cătălin Tomina, Ștefan Adrian Petruțiu, Bogdan Crișan, Daniel-Corneliu Leucuța, Cristian Mihail Dinu

**Affiliations:** 1 Department of Periodontology, Faculty of Dentistry, University of Medicine and Pharmacy Iuliu Hațieganu, 400012 Cluj-Napoca, Romania; tomina.darius@umfcluj.ro; 2 MedArtis Dent Private Dental Clinic, 400130 Cluj-Napoca, Romania; crisan.bogdan@umfcluj.ro (B.C.); cristian.dinu@umfcluj.ro (C.M.D.); 3 Department of Maxillofacial Surgery and Implantology, Faculty of Dentistry, University of Medicine and Pharmacy Iuliu Hațieganu, 400012 Cluj-Napoca, Romania; 4 Department of Medical Informatics and Biostatistics, Iuliu Hatieganu University of Medicine and Pharmacy, 400349 Cluj-Napoca, Romania; dleucuta@umfcluj.ro; 5 Department of Oral and Cranio-Maxillofacial Surgery, Faculty of Dentistry, University of Medicine and Pharmacy Iuliu Hațieganu, 400012 Cluj-Napoca, Romania

**Keywords:** dental implant, success rate, survival rate, periodontitis, implant prosthetic, bone level, multivariate analyses

## Abstract

The objective of the present study was to evaluate the 5-year outcome of dental implant therapy and assess the survival, success, and biological and technical complications. A periodontal and prosthetic-oriented evaluation was conducted on the variables that can influence the long-term predictability of implant therapy. A total of 615 patients and 1427 dental implants from a private clinic (MedArtis Dent, Cluj-Napoca, Romania) were enrolled in the study. The study was a longitudinal cohort with data collected retrospectively from physical/digital dental records in combination with a cross-sectional clinical/radiological examination. Results showed that periodontal diagnosis at baseline had a significant impact on the marginal bone loss prediction. Data showed that the cylindrical implant with an internal 45° medium taper connection experienced a higher rate of bone resorption compared to the tapered implant with the 5° internal connection. Screw-retained restorations and overdentures showed a statistical decrease in the marginal bone level height when compared to the cemented restorations. Data from our study suggest that not only is there a powerful association between recall visits and the rate of complications in dental implant treatment, but a lack of professional maintenance predicts a higher bone level loss during the 5-year interval.

## 1. Introduction

Nowadays, implant therapy represents a gold standard for treating edentulous single and multiple gaps up to full arch rehabilitation [1,2]. It has been more than 45 years since implant therapy was introduced as a method for replacing missed teeth [1,2]. Short- and long-term studies showed a significant increase in the quality of life for edentulous patients treated with implant-supported prostheses [3]. Over the years, many studies have shown a high survival rate for implants placed in edentulous jaws [2,4,5,6]. To objectively quantify the long-term predictability of implant therapy, the survival and success rates were evaluated over time [7,8]. The survival rate is defined as “implant and fixed prosthesis present in the mouth independent of biological and/or technical complications” [2,9,10]. Success, on the other hand, is defined by the same authors [9,10] as “implant and fixed prosthesis being free of all complications over the entire observation period”. These terms were unfortunately widely, and somehow improperly, used over time in different studies, making it impossible to extract evidence-based conclusions [2,7]. While the survival term ignores all the biological and esthetic complications that may occur during implant and implant-supported restoration functions, the success rate is more comprehensive, allowing a holistic appreciation of the outcomes of implant therapy [11]. Implant success depends mainly on observing the peri-implant health and prosthetic outcomes over time. Peri-implant health is clinically defined by the “absence of erythema, bleeding on probing, swelling and suppuration” [12,13,14]. The prevalence of peri-implant mucositis cited in the literature is somewhere between 14.2% to 38.6% depending on the applied criteria, while the prevalence of peri-implantitis was found to vary from 3.6% up to 4.7% when applying the same criteria for a functional period of 7–8 years [15]. A recent systematic review and meta-analyses showed that the prevalence of peri-implantitis at the patient level was 19.53%, while at the implant level, the same research showed a prevalence of the disease of 12.53% [16]. Other studies showed a higher prevalence, 56.6% at the patient level and 27.9% at the implant level, respectively [17]. This shows that peri-implant disease is highly prevalent among implant patients, but the lack of data uniformity is due to the absence of defined clinical criteria for the disease. Implant-supported prosthesis success can be defined by the presence of the prosthetic work in the mouth without any complications over the functioning period [18]. For a single implant retained crown, the overall 10-year survival rate of the implants was demonstrated in the studies to be 95.2% [4,18]. Yet, the survival rate of the crowns supported by the same implants was 89.4% [4,18].

For this reason, the need for a comprehensive evaluation of the long-term outcome of implant therapy is incurred. The absolute difference in the survival and success rates of implant therapy can be translated, at a patient level, as a decreased quality of life, second-stage surgical and prosthetic procedures, and higher costs. In dental implant research, a distinction should be made between the patient and implant site level. While patients are different from one another regarding anatomy, physiology, and local and general habits, it is reasonable to assume that sites within the same mouth are correlated within some limits [5]. However, complications of dental implant therapy occur. These complications can be biological, mechanical, technical, and esthetic. The biological complications are represented by early failures, mucositis that can further develop into peri-implantitis, and loss of osteointegration [19,20]. An objective parameter to observe and assess the biological outcomes of implant therapy is represented by marginal bone loss (MBL). This parameter, if measured at different time frames, can offer perspectives on implant treatment success that is dependent on the implant type, implant neck design, the level of submerging, the prosthetic connection, and the type of restoration and materials used [21].

Mechanical and technical complications are also known as prosthetic complications in implant therapy [18,22]. Implant fractures and abutment failures represent mechanical complications, while any complications related to the dental laboratory manufacturing process are placed under technical complications, such as fracture of the framework, fracture of the veneering materials, chipping, etc. [18,20,22]. Usually, mechanical and technical complications are addressed together under “technical complications” [2,18] and can be classified as major, medium, and minor [2,4]. The esthetic complications usually occur if the three-dimensional implant position is not properly managed and not prosthetically driven. Of course, soft tissue management plays an important role, as well as restoration materials [23,24,25].

All the biological and technical complications, if not treated, can lead to the failure of dental implant treatments. To minimize the incidence of complications, implant treatments should be meticulously planned; the implant insertion should be prosthetically guided with respect to all biological and physiological considerations in a perfect three-dimensional position. The prosthetician should carefully choose all the implant components and materials to be used, and last but not least, the patients should be included in a custom-made maintenance program after treatment, especially patients diagnosed and treated for periodontitis. During maintenance, the active sites should be diagnosed and addressed as recommended by the treatment guides [26], keeping in mind that treatment procedures to save the remaining affected teeth are evolving, offering an overall better prediction for the affected teeth and implants [27,28].

The objective of the present study was to evaluate the 5-year outcome of dental implant therapy and assess survival, success, and biological and technical complications. Moreover, the study aimed to identify periodontal and prosthetic risk indicators at the patient and implant level with a significant impact on the incidence of bone resorption and bone remodeling around dental implants. For this reason, we tested the following null hypothesis: there is no association between periodontal background and type of prosthetic work and the marginal bone loss on a 5-year follow-up in implant therapy.

## 2. Materials and Methods

### 2.1. Study Design

**Figure 1:**
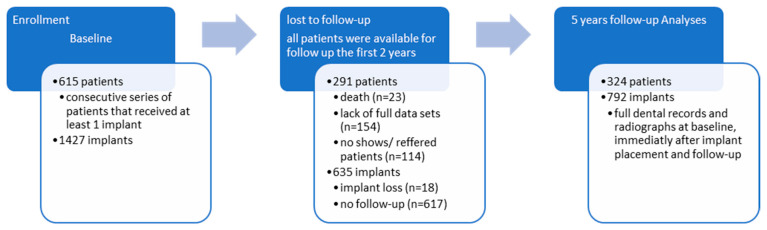
Study design flow diagram.

This is a longitudinal cohort study with data collected retrospectively from physical and digital dental records in combination with a cross-sectional clinical and radiological examination, as shown in Figure 1. A consecutive series of patients was observed in our study.

### 2.2. Setting

All patients were referred to a specialist implantology clinic (Med Artis Dent, a private dental clinic in Cluj-Napoca, Romania), where implant therapy was performed from 2015 to 2016. The follow-up time was five years in a population consisting of a selected group of patients with dental implants.

### 2.3. Participants

All the patients that received at least one dental implant from 1 January 2015 up to 31 December 2016 were included in the study group regardless of the number of implants placed and their anamnestic and clinical background. The dental implant surgical procedures were performed by two maxillo-facial surgeons, whereas prosthodontists or general dentists performed the prosthetic implant treatment, and two specialized periodontists evaluated the implant treatment outcomes over the years.

The included subjects were 615 patients with a total number of 1427 dental implants. The anamnestic, clinical, and radiographic variables at baseline (i.e., the clinical examination, including the treatment plan for implant therapy) and following dental visits to the specialist clinic were obtained from physical and digital dental records. All patients were available for follow-up in the first two years. However, 291 patients and 635 implants were excluded from the study because they did not meet the inclusion criteria: full dental records, including the full dental status, a complete diagnostic and treatment plan, and radiologic examinations both at baseline, immediately after implant insertion, and at follow-up. The final sample included 324 patients and 792 implants.

### 2.4. Variables, Data Sources, and Measurement

The following variables were registered from the dental records by the same two investigators at baseline.

Anamnestic variables: age, sex, smoking habits (yes/no and number of cigarettes per day), general health, diabetes (yes/no, type), cardiovascular disease (yes/no), osteoporosis (yes/no), periodontal/prosthetic diagnosis at baseline (edentulous/periodontal health/stage1, stage2, stage3, stage4 periodontitis, according to the latest European Federation of Periodontology and American Association of Periodontology classification of periodontal and peri-implant diseases [29]), periodontal treatment before dental implant therapy (no, yes), number of natural teeth at baseline, and prosthetic treatment performed by a prosthodontic specialist (no, yes).

Clinical variables: number of teeth; history of treated periodontitis Stage I–IV (no, yes = more than 30% of sites with periodontal pockets ≥ 4 mm BOP—bleeding on probing—and marginal bone loss > 3 mm) before implant treatment; the marginal alveolar bone level on digital dental radiographs of implants and teeth at baseline (baseline = implant installation up to prosthetic installation) was estimated using Planmeca Romexis^®^ (Planmeca Oy, Helsinki, Finland) dental imaging software. A calibration tool provided in the software program was used to calculate the distances in millimeters on panoramic radiographs or CBCT scans at teeth and implant levels, number of implants installed, number of prosthetic units per implant, implant position (maxillary/mandibular, anterior/posterior region), implant brand: MegaGen Any Ridge implants (MegaGen group, Daegu, Republic of Korea) and Bego Semados S implant system (BEGO Implant Systems GmbH & Co. KG, Bremen, Germany), implant dimension, bone augmentation procedures before implant treatment (no, yes, type—iliac crest/guided bone regeneration/onlay graft/socket preservation/sinus lifting), bone augmentation during implant surgery (no, yes, type—onlay graft/socket preservation/contour augmentation/sinus lifting/socket shield/ridge split), healing (open, closed), implant loading (conventional, early, immediate), soft tissue grafting (yes/no, type—free gingival graft/connective tissue graft), and prosthetic design (cemented/screw-retained. overdenture). The following variables were registered from the dental records at follow-up dental visits by investigators, preceded by calibration according to clinical measurements for these two experienced periodontists: biological complications (year, type, treatment), technical complications (year, type, treatment), follow-up at a specialist clinic (no, yes = regular check-ups yearly by periodontist or prosthodontist), and regular supportive care at the periodontal clinic (no, yes = regular maintenance therapy by a periodontist at the specialist clinic at least twice a year); marginal bone levels were estimated using Planmeca Romexis^®^ dental imaging software by measuring the distance between implant shoulder and the first bone to implant contact. The most unfavorable mesial/distal measurement was registered. All patients were available for follow-up in the first two years.

Biological complications: classified as peri-implant mucositis, peri-implantitis, or absence of osteointegration. The definition of mucositis was bleeding on pocket probing without radiographic progressive bone loss. Peri-implantitis was defined as bleeding/suppuration on probing, a pocket depth > 4 mm, and progressive radiographic bone loss ≥ 2 mm compared to the baseline radiographic bone level.

Technical complications were divided into three categories:

- Minor: easily handled chairside (loss of retention or de-bonding of implant crown(s), fracture of porcelain on implant crown(s), loss of screw hole sealing, or abutment screw loosening).
- Medium: manageable but at a greater cost and chair time required (fracture of abutment screw or abutment screw loosening).
- Major: new construction required or major repairs with even greater costs and a substantial amount of chair time (fracture of porcelain on implant crown(s), implant fracture, abutment screw loosening, or fracture of prosthesis).
- Survival/success of implants was divided into two categories:
  - Survival: the implant exists in the mouth.
  - Success: an implant without biological and/or technical complications.

### 2.5. Statistical Analyses

The unit of analysis was the patient or the implant, as specified per analysis. Numbers and percentages were used to describe the qualitative data. Medians and interquartile ranges were used to describe non-normally distributed quantitative data. Comparisons between independent groups concerning the qualitative data were performed with the chi-squared test or Fisher exact test (in the case of low expected frequencies). Comparisons between independent groups concerning non-normally distributed data were performed with the Wilcoxon rank-sum test (for two groups). The simple comparisons per implant do not take into account the correlation related to the patient. Survival data was presented with Kaplan-Meyer graphs. A multiple Cox regression, clustered by the patient (several implants per patient), was fit with manually specified predictors based on clinical experience loading (early or immediate vs. conventional). The Cox proportional hazard assumption was checked with a statistical test. The multicollinearity assumption was checked with variance inflation factors. The small number of events prevented us from using more predictors to avoid overfitting. The model was presented as hazard ratios with 95% confidence intervals and p-values. A generalized linear mixed effects regression model was fit to predict implant success rate, taking into account the correlation induced by multiple implants per patient, having as independent variables that were manually specified by clinical experience, rationale, and literature: gender, osteoporosis, periodontal diagnosis baseline/grouped (stage 1, 2 or 3, 4 vs. healthy, edentulous), implant type (MEGAGEN vs. BEGO), prosthetic (OD, or screw-retained vs. cemented), implant position (posterior mandible, anterior maxilla, posterior maxilla, or anterior mandible), loading (early, immediate, or delayed), and recall at a specialized clinic. The important number of implants analyzed allowed us to use an important number of predictors in this model without breaking the rule of 10 observations per variable. The model was presented as coefficients with 95% confidence intervals and p-values. All statistical tests were two-tailed with a significance level of 0.05. Statistical analysis was performed with the R environment for statistical computing and graphics (R Foundation for Statistical Computing, Vienna, Austria), version 4.1.2 [R Core Team. R: A Language and the Environment for Statistical Computing, R Foundation for Statistical Computing: Vienna, Austria; 2014].

## 3. Results

### 3.1. Patient Demographics

Statistical analyses focused on 615 patients and 1427 implants, with an average of 2.32 implants per patient. Among the 615 patients with the 1427 implants inserted, the 5-year data regarding the examined variables were available for only 324 patients and 792 implants, recalculating the average number of implants per patient at 2.44. The 47.31 percent drop-out rate was due to illness, death, or change of medical service provider. The majority of patients that failed to attend the recall visits were patients referred to our clinic for the only purpose of implant therapy, while the restorations and follow-ups were assumed to be performed by the general practitioners that referred the patient. The frequency distribution of the patients by age, gender, smoking status, and general state of health can be observed in Table 1. The mean age of the patients at follow-up was 45 (range 17–79), and 25.31% were smokers, while 23.46% had a frequency of more than ten cigarettes per day. The follow-up period on which the assessment for this study was made was 60 months. At the beginning of implant therapy, a thorough periodontal examination was made, and staging was performed retrospectively at the follow-up using the prior periodontal diagnostic correlated with the clinical and self-reported symptoms and radiological data. Out of the total number of 324 patients, 88.58% were included in one of the four periodontitis stages, while 45.99% of the patients suffered from Stages 3 and 4 periodontitis. Of the included subjects at follow-up, 59.57% received specific periodontal treatment before implant surgery.

### 3.2. Implant and Surgical Site Characteristics

Two implant systems were used in this study: the MegaGen Any Ridge implant (MegaGen Group, Daegu, Republic of Korea) and the Bego Semados S implant system (BEGO Implant Systems GmbH & Co. KG, Bremen, Germany), as shown in Table 2. The implant dimensions are shown in Table 3. The combining implant position sites were used according to Buser’s classification [30]. Before and during implant placement, 65.28% of the implants received bone augmentation procedures in accordance with the implant site, as shown in Table 2. Out of the 792 implants evaluated at follow-up, almost 29% were left with a healing abutment after insertion, and 71.21% were conventionally loaded. The percentage of implants that received soft tissue grafting was 16.80%, while over 66% of the implants received post-care periodontal and prosthetic procedures in specialized clinics. All types of restorations were considered in this study, from single units to full arch implant rehabilitations. Due to the large number of implants, we resorted the evaluations to the number of prosthetic units per implant and the restoration type, cemented, screw-retained, or overdenture, as shown in Table 2. Mainly, the material and technique used for the prosthetic restorations were porcelain fused to metal and veneered zirconia. All of the cemented restorations were fabricated on customized titanium or zirconia abutments, while the removable overdentures were telescopic prostheses on galvanized gold telescopes.

### 3.3. Implant Survival and Success Rate

Out of the 792 implants evaluated after 60 months of function, 784 implants were still present in the patient’s mouth, leading to a survival rate of 98.99%, while the implant success rate was 91.92% (Table 4).

#### 3.3.1. Survival Rate

After the statistical analyses, some associations were observed, as shown in Table 5. All of the failed implants were placed in heavily smoking patients suffering from stages 3 and 4 periodontitis that was not previously treated or treated by means of full tooth extraction therapy with open healing after implant insertion. Of the failed implants, 62.5% were loaded immediately.

The results of the Cox multivariate analyses predicting the implants’ survival rate, taking into account the correlated observations, are presented in Table 6.

**Figure 2:**
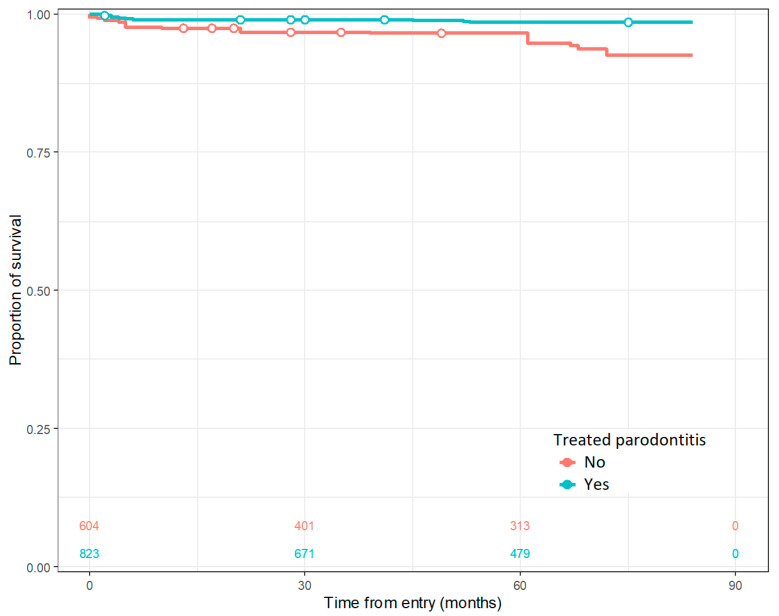
Cumulative survival rate for treated and untreated periodontitis.

The only factor, out of the ones tested in the regression model, that predicts the implant survival rate is the previously applied treatment for periodontitis, as shown in Figure 2.

#### 3.3.2. Success Rate

Implant treatment was considered successful when the implant was still present in the patient’s mouth without biological or mechanical complications at follow-up. The success rate for this study was 91.92%. The biological complications that occurred during the implant treatment function were less than 4% (Table 7). Even though the biological complications rate was low, the clinical significance is of concern. Besides the peri-implant mucositis, all other biological complications (lack of osteointegration and peri-implantitis) needed a second surgery or implant removal. Technical complications of implant treatment occurred more often, with a rate of almost 6%. Of the 41 implants that suffered this complication, 27 were minor, easily handled chairside. Ten implants, out of 792, experienced major complications during the five years of function and needed prosthetic work replacement (Table 7).

Regarding the evaluated parameters, we found a statistically significant association between the implant success rate and severe periodontitis (Table 8). Even if the disease was stabilized and contained, biological complications occurred in 65.38% of implants in patients suffering from Stages 3 and 4 periodontitis. Another significant association was found in the number of prosthetic units per implant. Of the implants that presented biological or technical complications, 95.31% had a ratio of more than three prosthetic units per implant (Table 8).

All the patients and implants evaluated were available for a two-year follow-up. The parameters that influenced the implant success rate for this time frame, including all implants (n = 1427), are presented in Table 9.

The associations between the implant success rate and evaluated variables, including one implant per patient, are shown in Table 10. In the table, the implant success rate is reported at a patient level, presenting only the statistically significant associations.

### 3.4. Marginal Bone Level

The level of marginal bone loss was assessed by site-specific measurements and registered at follow-up as the difference from baseline. All associations with the evaluated parameters refer only to the assessed implants at follow-up, at least 60 months after placement. We found important associations regarding marginal bone loss during implant function, as seen in Table 10. Stage 3 and 4 periodontal patients suffered from accelerated bone loss during implant function, regardless of the stabilization of the disease. Bego Semados S implants had a statistically significant difference in bone remodeling compared to MegaGen AnyRidge dental implants. Screw-retained restorations, as well, predicted a greater marginal bone loss than cemented ones. The anterior maxilla and mandible showed a greater difference in marginal bone loss in comparison to posterior areas, while immediate and early loading showed the same pattern. Implants that were evaluated and maintained in specific specialized clinics had better outcomes in terms of bone remodeling (Table 11).

**Figure 3:**
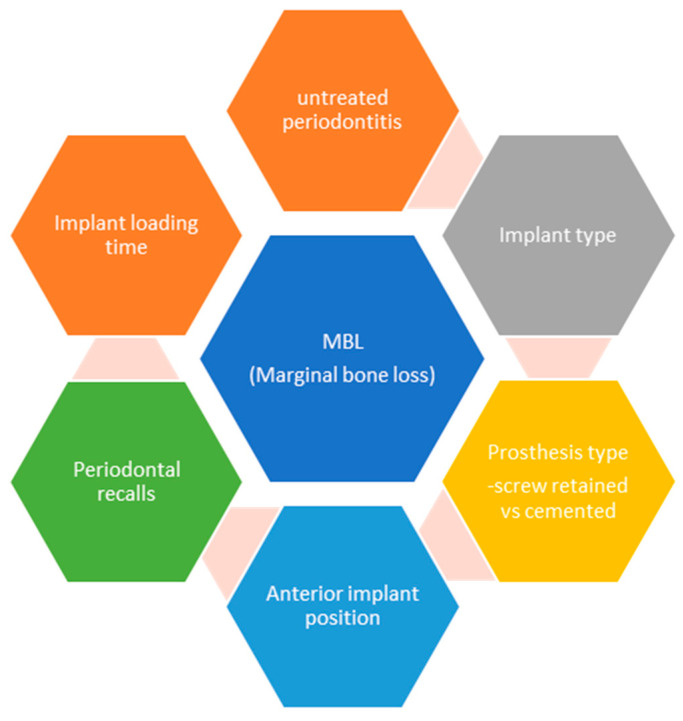
Predicting factors for marginal bone loss in implant therapy (summarized).

As we can see in the generalized linear regression model with mixed effects on all implants predicting the level of marginal bone level (Table 12), the periodontal diagnosis at baseline, the implant type, the type of prosthetic work, and specialized recalls are statistically significant predicting factors for the marginal bone level rate of remodeling (Figure 3).

## 4. Discussion

Whenever choosing or proposing an implant-supported treatment, the clinician should carefully select the cases, plan the treatment, supervise, and maintain the results. It is essential to adapt the treatment plan, the materials used, and the design of choice, relying on emerging and trustful research with at least 5-year follow-ups or even longer. In most cases, the patient is unaware of the risks and complications of implant treatment [31]. For this reason, patient perception should be taken into consideration when evaluating implant therapy outcomes [31,32,33], given that patients expect this treatment to last a long time, regardless of the reason for tooth loss or oral health status [2,34]. The survival and success rates are strictly dependent on the mechanical and biological complications that can occur, from implant insertion up to implant restoration function in the oral cavity. This can be correlated with the healing type, soft tissue augmentation, type of prosthetic design, number of prosthetic units per implant, implant loading frame, and last but not least, the proper professional maintenance of the implant support restoration.

In this study, in the selected patient group, the survival rate at the implant level after five years of function was 98.99%. One of the first systematic reviews and meta-analyses, where the authors included prospective longitudinal studies with a follow-up time of at least five years, reporting on the survival rate of dental implants, was published by Berglundh et al. [35]. The authors determined the implant survival rate on fixed vs. mobilized dental prosthetics and concluded that the 5-year survival rate of implants supporting fixed reconstructions was 95%, while the survival rate of implants supported overdentures was 92%. More recent studies [4,20,36] that evaluated the survival rate of implants reported a 10-year survival rate of 95.2% for implant-supported single crowns [36], 93.1% for implants supporting fixed dental prostheses [4], and 82.1% for implants supporting combined tooth-implant-supported prostheses [37]. The reasons for a higher survival rate for our study could be (1) the standardization of the surgical and prosthetic procedures that were performed by the same surgeons and prostheticians, (2) the use of the same dental implant systems, (3) proper surgical management of the implant site, or (4) the technological and scientific progress of the last decade. Moreover, the factors that influence the survival rate at a patient level should be taken into consideration. The only exclusion criteria for the patient cohort involved in our study was the absence of clinical and radiological data at both baseline and follow-up. For a better resemblance to the general population and for research purposes, patients suffering from general health problems (diabetes, osteoporosis, or heart failure), smokers, a history of periodontal disease, and lack of sufficient alveolar bone were included and counted in our study. The majority of the implants used for this study were regular-length implants, while the surgical procedure was performed via a two-step approach in many cases. There are short-term studies that show a similar success rate when replacing a regular-length implant with two short implants avoiding the second-stage surgery [38]. This could be a valid option, but further research and longer-term studies are needed to properly evaluate the therapy outcome.

The implant success rate for the present study was found to be 91.92% after 60 months of function. Data in the literature varies from 34% to 100% success rates [2,6,36,39,40]. This difference can reflect an evaluation bias regarding the complications. The objective criteria for diagnosing mucositis and peri-implantitis used in this study were those established in the consensus report on peri-implant diseases and conditions of the 2017 World Workshop [13]. The frequency of peri-implantitis for our study was 1.26%, while the prevalence of mucositis was under 0.5%. In studies with almost the same diagnostic criteria and a minimum of five years of follow-up, the prevalence of peri-implantitis at the patient level varied from 14.5% to 37% [2,41,42,43]. The difference could be again attributed to the data uniformity. A very important thing to consider is that most studies in the literature consider the prevalence of periodontitis at the patient level, while our study refers to the prevalence at the implant level; when we refer to implant success rate, data should not be reported at the patient level. From a clinical perspective, it often happens to diagnose a peri-implant site infection on one or a limited number of implants within the same mouth. However, the lack of data reporting uniformity can be misleading because these complications are usually a combination of biological, technical, and esthetic problems. Additionally, the majority of papers report their outcomes on a single brand of implant, which can restrict their conclusions to a specific implant type and design. An interesting paper published by Pjetursson et al. [39] compared the data regarding the survival and success rates of implant therapy in publications before and after the year 2000; the purpose of his paper was to establish if there had been significant improvement in implant dentistry during the decade. The authors reported that there had been a significant decrease in many of the complications, but their incidence was still high [39]. Analyzing this data stresses the importance of identifying and preventing these complications from happening. Still, it has been 20 years since this paper was published, and there is no doubt regarding the evolution of implant treatment over the last two decades.

In this study, as observed in many other studies, smoking and having a background of periodontitis significantly affected the implant’s success and survival rate. While the implant survival rate was directly associated with smoking, all the patients that lost their implants during the five years of function were heavy smokers (more than ten cigarettes per day*, p* = *0.006*). We did not find a statistically significant association between smoking and success rate at the implant level. A systematic review and meta-analysis on smoking and the risk of peri-implantitis failed to reveal that smoking was a risk factor at the patient level [44,45]. This could again represent a risk of bias due to the lack of uniformity in reporting. While we count other variables for the implant success rate, the results can be somehow influenced by the input of data. A more recent systematic review and meta-analysis concluded that “Implants placed in smokers present a 140.2% higher risk of failure than implants placed in nonsmokers” [45]. If we take a closer look at our data and evaluate the global success rate, including the implants that did not meet the inclusion criteria (all the patients were available for follow-up at two years), we can see that 41.27% of the patients with complications were smokers (*p* < *0.001*), while 40.94% smoked more than ten cigarettes per day (*p* < *0.001*). We can conclude from this finding that the association between smoking and implant success rate was not so powerful in our study because of the drop-out rate and the lack of data at follow-up. Therefore, there is a need for prospective studies in order to draw final conclusions on the topic.

However, a non-disputable variable that influenced the outcomes of the survival and success rates in this study was periodontal diagnosis at baseline. All the failed implants (the ones that did not survive) were placed in patients suffering from Stages 3 and 4 periodontitis without proper treatment or treatment by means of full extraction therapy. Regarding influence on the success rate, all the implants that experienced biological complications during functioning were placed in patients with Stages 3 and 4 periodontitis, regardless of treatment or specialized follow-up. There are studies demonstrating that periodontitis progression is significantly lower along regular compliers [46]. At an implant level, we could not find any association between regular supportive care and implant success rate. On the other hand, at a patient level, which is more suitable to predict a variable for this association, 68.5% of the patients with complications failed to present at their annual recalls at the specialized clinic (*p* < *0.001*), while 65.35% of the subjects diagnosed with a form of periodontitis failed to do their bi-annual periodontal check-ups. From another perspective, the group that obtained supportive care at the specialist clinic potentially reduced the rate of late implant loss but at the same time increased the complication reports, causing a detection bias.

Peri-implant bone stability is a major concern regarding implant treatment [47]. It has been proven that crestal bone stability is critical for soft tissue stability and the long-term success of implant treatment [48,49]. The implant abutment interface [50], the amount of keratinized mucosa [51], the soft tissue thickness [52], and the abutment height [53,54] have a direct impact on implant marginal bone loss. Out of the variables evaluated in our study, during the five years of function, and after applying a generalized linear regression predicting the marginal bone level, we found that:

1. Periodontal diagnosis at baseline had a significant impact on the level of marginal bone loss prediction. Regardless of treatment, Stage 3 and 4 periodontitis patients experienced a higher level of marginal bone loss in comparison to Stages 1 and 2. A comprehensive review of articles published over 42 years concluded that “There is an increased risk of peri-implantitis in smokers compared with non-smokers (reported odds ratios from 3.6 to 4.6). The combination of a history of treated periodontitis and smoking increases the risk of implant failure and peri-implant bone loss” [55].
2. For the implants researched in our study, data show that the cylindrical implant with an internal connection of a 45° medium taper (Bego Semados S implant system BEGO Implant Systems GmbH & Co. KG, Bremen, Germany) experienced a higher rate of bone resorption compared to the tapered implant with the 5° internal connection (MegaGen Any Ridge implant, MegaGen group, Daegu, Korea). Both implant systems have a rough surface and a machined polished neck. Although the studies in the literature are inconclusive regarding the survival/success rate of different implant types (tapered vs. cylindrical), the connection type seems to have an impact on long-term stability, especially the morse-taper connection [56]. The primary stability of tapered implants has been demonstrated to be higher than that of cylindrical ones [57], which can have a direct impact on the marginal bone level if loaded immediately [58]. Even though our findings are similar to other studies, we cannot neglect the risk of bias due to the large difference in implant numbers on the two used systems and the observational nature of our study.
3. The type of prosthetic work showed a significant prediction of marginal bone loss. Screw-retained restorations and over dentures showed a statistical decrease in the marginal bone level height when compared to the cemented restorations. In the literature, there is still a debate regarding cemented vs. screw-retained implant restorations. With the use of stock abutments in the past, a risk for developing peri-implantitis due to improper excess removal after cementation was demonstrated [59,60]. As a mechanical complication, screw loosening happened more often for the screw-retained ones than for the cemented abutments [61]. A recent systematic review and meta-analysis demonstrated that “multiple abutments disconnections significantly affected marginal bone loss changes in partially edentulous patients” [62]. With the use of standardized titanium-based implant connections and customized implant abutments, we can nowadays make cemented prosthetic restorations that overcome the drawbacks from the past and best preserve the marginal bone level. The cemented restorations used in this study all followed these principles in accordance with other research [63].
4. Even though other studies reported the lack of evidence for the association between recall visits and the rate of complications in dental implant treatment [2], data from our study suggest that not only is there a powerful association between these two variables, but a lack of professional maintenance predicts a higher bone level loss during the 5-year functional period.

## 5. Conclusions

### 5.1. Limitations and Strengths

The present study is subject to potential biases due to its observational and retrospective nature. Cause-effect relations cannot be demonstrated with certainty, only associations. Confounding is an important issue. Nevertheless, our multivariate models included the maximum number of variables to control for confounders but to prevent overfitting at the same time.

Our study has important strengths, especially regarding the large cohort of patients and a large number of implants, as well as the long follow-up (five years). Moreover, the multivariate analyses, keeping into account multiple confounders, help get closer to the truth. Finally, we used specialized methods in the multivariate analyses to take into account the correlation effects of having several implants per patient.

### 5.2. Implications for Practice

Despite the limitations of this retrospective study, the results confirm the necessity of using standardized terms when referring to implant treatment outcomes. The term survival should be replaced with the term success, where all complications should be registered, as well as implant early, delayed, or late loss as a consequence of a biological or mechanical complication. The practitioners should be well aware of the possible complications of implant therapy, which should be discussed with the patient before treatment, and establish ground rules regarding the treatment expectations.

Periodontitis, even treated, predicts a higher marginal bone level loss over time. That could be due to the general pattern of resorption and remodeling, not necessarily resembling the onset of periodontitis. Periodontal diagnostic should dictate the treatment plan, as well as the level of implant submergence, keeping in mind a faster resorption rate for these patients. The prosthetic components should be carefully selected in order to avoid, as much as possible, marginal bone level loss. Awareness of the need for professional recalls after treatment should be raised even if this awareness would probably reduce the implant success rate due to the potential diagnosis of minor technical or biological complications and would definitely reduce the implant loss rate over time.

Within the limitations of the present study, the baseline diagnosis of periodontitis can predict and influence the long-term survival and success of implant therapy, and the null hypothesis was rejected. Cemented prosthetic restorations showed better integration and slower marginal bone remodeling rate compared to the screw-retained ones. Smoking has a negative impact on implant survival and success rates when evaluated at a patient level. Even if it can negatively influence the success rate, specialized follow-up can have a major impact on the long-term implant survival rate and quality of life maintenance.

## Figures and Tables

**Table 1:** Patients characteristics.

Characteristic	All Patients (n = 615)	Patients with Implants Surviving ≥ 60 Months (n = 324)
Age, median (IQR) [range]	45 (38–57) [17–83]	45 (38–57) [17–79]
Gender (F vs. M)	359/615 (58.37)	185/324 (57.1)
Smoker, n (%)	162/615 (26.34)	82/324 (25.31)
No of cigarettes/day, median (IQR)	0 (0–5) [0–30]	0 (0–0) [0–30]
No of cigarettes/day ≥ 10, n (%)	148/615 (24.07)	76/324 (23.46)
Diabetes (total), n (%)	14/615 (2.28)	5/324 (1.54)
Diabetes, n (%)		
No:	601/615 (97.72)	319/324 (98.46)
Type1:	1/615 (0.16)	0/324 (0)
Type2:	13/615 (2.11)	5/324 (1.54)
Cardiovascular disease, n (%)	29/615 (4.72)	21/324 (6.48)
Osteoporosis, n (%)	46/615 (7.48)	20/324 (6.17)
Periodontal diagnosis at baseline, n (%)		
Edentulous:	13/615 (2.11)	8/324 (2.47)
Health:	49/615 (7.97)	29/324 (8.95)
Stage 1:	109/615 (17.72)	48/324 (14.81)
Stage 2:	170/615 (27.64)	90/324 (27.78)
Stage 3:	158/615 (25.69)	83/324 (25.62)
Stage 4:	116/615 (18.86)	66/324 (20.37)
Treated periodontitis, n (%)	348/615 (56.59)	193/324 (59.57)
No. of natural teeth at baseline, median (IQR) [range]	20 (15–24) [0–31]	19.5 (16–24.25) [0–31]
No. of natural teeth at recall, median (IQR) [range]	19 (14–24) [0–31]	19 (13–24) [0–31]
No. of inserted dental implants, median (IQR) [range]	2 (1–3) [1–22]	2 (1–3) [1–8]
No. of prosthetic units/implant ≤ 3, n (%)	610/615 (99.19)	321/324 (99.07)

IQR, interquartile range.

**Table 2:** Implant characteristics.

Characteristic	All Implants (n = 1427)	Implants Surviving ≥ 60 Months (n = 792)
Implant, n (%)		
MEGAGEN	1237/1427 (86.69)	716/792 (90.4)
BEGO	190/1427 (13.31)	76/792 (9.6)
Prosthetics, n (%)		
Cemented:	522/1427 (36.58)	275/792 (34.72)
OD:	199/1427 (13.95)	114/792 (14.39)
Screw:	706/1427 (49.47)	403/792 (50.88)
Implant position, n (%)		
Mandibular anterior:	87/1427 (6.1)	53/792 (6.69)
Mandibular posterior:	559/1427 (39.17)	295/792 (37.25)
Maxillary anterior:	208/1427 (14.58)	124/792 (15.66)
Maxillary posterior:	573/1427 (40.15)	320/792 (40.4)
Bone reconstruction before implant therapy, n (%)		
Iliac crest:	18/1425 (1.26)	9/791 (1.14)
GBR:	13/1425 (0.91)	11/791 (1.39)
No:	1100/1425 (77.19)	616/791 (77.88)
Onlay graft:	91/1425 (6.39)	43/791 (5.44)
Alveolar grafting:	66/1425 (4.63)	41/791 (5.18)
Buccal reconstruction:	10/1425 (0.7)	5/791 (0.63)
Sinus lift:	127/1425 (8.91)	66/791 (8.34)
Bone reconstruction during implant therapy, n (%)		
No:	748/1427 (52.42)	421/792 (53.16)
Onlay graft:	2/1427 (0.14)	0/792 (0)
Alveolar grafting:	107/1427 (7.5)	66/792 (8.33)
Buccal reconstruction:	460/1427 (32.24)	249/792 (31.44)
Sinus lift:	77/1427 (5.4)	38/792 (4.8)
Socket shield:	3/1427 (0.21)	3/792 (0.38)
Splitting:	30/1427 (2.1)	15/792 (1.89)
Bone reconstruction all (before and during implant therapy), n (%)	949/1427 (66.5)	517/792 (65.28)
Healing (Open vs. Closed), n (%)	372/1427 (26.07)	228/792 (28.79)
Free gingival graft, n (%)	103/1427 (7.22)	60/792 (7.58)
Connective tissue graft, n (%)	130/1427 (9.11)	73/792 (9.22)
Loading, n (%)		
Conventional:	1079/1427 (75.61)	564/792 (71.21)
Early:	161/1427 (11.28)	109/792 (13.76)
Immediate:	187/1427 (13.1)	119/792 (15.03)
Recall at a specialized clinic, n (%)	718/1427 (50.32)	541/792 (68.31)
Periodontal check-up at specialized clinic, n (%)	683/1427 (47.86)	525/792 (66.29)
Implant loss (early/late), n (%)	37/1427 (2.59)	8/792 (1.01)
No. of prosthetic units/implant, median (IQR) [range]	1 (1–1.5) [1–6]	1 (1–1.5) [1–6]
Implant survival time (months), median (IQR) [range]	60 (30–67) [0–84]	66.5 (62.75–72) [60–84]

IQR, interquartile range; OD, overdentures; GBR, guided bone regeneration.

**Table 3:** Implant dimensions.

Implant Diameter (mm)	Implant Length	No of Implants (%) n = 792
3	11.5 ± 0.84	10 (1.26)
3.25	10.75 ± 1.67	15 (1.89)
3.5	10 ± 2.12	302 (38.13)
3.75	11.5 ± 1.22	49 (6.18)
4	10.75 ± 2.68	284 (35.85)
4.1	12.25 ± 0.75	7 (0.88)
4.5	10.75 ± 2.68	95 (11.99)
5	10 ± 2.12	21 (2.65)
5.5	10.75 ± 1.67	9 (1.13)

Data are presented as mean ± standard deviation.

**Table 4:** Implant survival and success rate.

Characteristic	All Implants (n = 1427)	Implants Surviving ≥ 60 Months (n = 792)
Implant survival, n (%)	1390/1427 (97.41)	784/792 (98.99)
Implant success rate, n (%)	1300/1427 (91.1)	728/792 (91.92)

**Table 5:** Implant survival rate associated with tested variables at follow-up.

Implant Survival Rate:	Yes (n = 784)	No (n = 8)	*p*-Value
Sex (F vs. M), no (%)	445 (56.76)	2 (25)	0.085
Smoker, no (%)	215 (27.42)	6 (75)	*0.007*
No. of cigarettes/day ≥ 10, no (%)	205 (26.15)	6 (75)	*0.006*
Diabetes, no (%)	18 (2.3)	0 (0)	1
Osteoporosis, no (%)	58 (7.4)	0 (0)	1
Periodontal diagnosis at baseline, no (%)			0.234
Stage 1:	82 (11.47)	0 (0)	
Stage 2:	193 (26.99)	0 (0)	
Stage 3:	210 (29.37)	4 (66.67)	
Stage 4:	230 (32.17)	2 (33.33)	
Treated periodontitis, no (%)	479 (61.1)	0 (0)	*<0.001*
Periodontal diagnosis at baseline and treatment no (%)			*<0.001*
Stage 1,2 untreated:	91 (12.73)	0 (0)	
Stage 1,2 treated:	184 (25.73)	0 (0)	
Stage 3,4 untreated:	145 (20.28)	6 (100)	
Stage 3,4 treated:	295 (41.26)	0 (0)	
Implant type (BEGO vs. MEGAGEN), no (%)	76 (9.69)	0 (0)	1
Prosthetics, no (%)			0.065
Cemented:	275 (35.08)	0 (0)	
OD:	112 (14.29)	2 (25)	
Screw:	397 (50.64)	6 (75)	
Loading no (%)			*<0.001*
Conventional:	563 (71.81)	1 (12.5)	
Early:	107 (13.65)	2 (25)	
Immediate:	114 (14.54)	5 (62.5)	
No of prosthetic units/implant ≤ 3, no (%)	775 (98.85)	8 (100)	1
Implant position, no (%)			0.213
Mandibular anterior:	53 (6.76)	0 (0)	
Mandibular posterior:	294 (37.5)	1 (12.5)	
Maxillary anterior:	121 (15.43)	3 (37.5)	
Maxillary posterior:	316 (40.31)	4 (50)	
Bone reconstruction before implant placement, no (%)			0.674
Iliac crest:	9 (1.15)	0 (0)	
GBR:	11 (1.4)	0 (0)	
No:	609 (77.78)	7 (87.5)	
Onlay graft:	43 (5.49)	0 (0)	
Alveolar grafting:	40 (5.11)	1 (12.5)	
Buccal reconstruction:	5 (0.64)	0 (0)	
Sinus lift:	66 (8.43)	0 (0)	
Bone reconstruction and implant placement, no (%)			0.184
No:	418 (53.32)	3 (37.5)	
Onlay graft:	0 (0)	0 (0)	
Alveolar grafting:	66 (8.42)	0 (0)	
Buccal reconstruction:	246 (31.38)	3 (37.5)	
Sinus lift:	36 (4.59)	2 (25)	
Socket shield:	3 (0.38)	0 (0)	
Splitting:	15 (1.91)	0 (0)	
Free gingival graft, no (%)	60 (7.65)	0 (0)	1
Connective tissue graft, no (%)	73 (9.31)	0 (0)	1
Healing (Open vs. close), no (%)	220 (28.06)	8 (100)	*<0.001*
Recall at a specialized clinic, no (%)	537 (68.49)	4 (50)	0.272
Periodontal check-up at specialized clinic, no (%)	521 (66.45)	4 (50)	0.453

OD, overdenture; GBR, guided bone regeneration.

**Table 6:** Multivariate Cox proportional survival model for implant survival predicted by loading, previously treated periodontitis, and bone reconstruction, clustered by patient.

Characteristic	HR ^1^	95% CI ^2^	*p*-Value
Loading			
Early vs. conventional	1.57	0.50, 4.97	0.4
Immediate vs. conventional	1.74	0.46, 6.65	0.4
Previously treated periodontitis	0.29	0.12, 0.73	*0.008*
Bone reconstruction (total vs. none)	1.11	0.43, 2.87	0.8

^1^ Hazard Ratio; ^2^ Confidence Interval.

**Table 7:** Biological and technical complications.

Characteristic	All Implants (n = 1427)	Implants Surviving ≥ 60 Months (n = 792)
Technical complication 1		
Absent:	1361/1427 (95.37)	751/792 (94.82)
Major:	11/1427 (0.77)	10/792 (1.26)
Medium:	6/1427 (0.42)	4/792 (0.51)
Minor:	49/1427 (3.43)	27/792 (3.41)
Technical complication 2 (Minor vs. Absent)	9/1427 (0.63)	9/792 (1.14)
Technical complication 3 (Minor vs. Absent)	2/1427 (0.14)	2/792 (0.25)
Biological complications		
Absent:	1392/1427 (97.55)	772/792 (97.47)
Mucositis:	3/1427 (0.21)	2/792 (0.25)
No integration:	18/1427 (1.26)	8/792 (1.01)
Periimplantitis:	14/1427 (0.98)	10/792 (1.26)

**Table 8:** Implant success rate associated with tested variables at follow-up (60 months).

Implant Success Rate:	Yes (n = 728)	No (n = 64)	*p*-Value
Sex (F vs. M), no (%)	417 (57.28)	30 (46.88)	0.107
Smoker, no (%)	199 (27.34)	22 (34.38)	0.229
No. of cigarettes/day ≥ 10, no (%)	190 (26.1)	21 (32.81)	0.244
Diabetes, no (%)	16 (2.2)	2 (3.12)	0.651
Osteoporosis, no (%)	55 (7.55)	3 (4.69)	0.615
Periodontal diagnosis at baseline, no (%)			*0.018*
Stage 1:	78 (11.66)	4 (7.69)	
Stage 2:	179 (26.76)	14 (26.92)	
Stage 3:	206 (30.79)	8 (15.38)	
Stage 4:	206 (30.79)	26 (50)	
Periodontal treatment, no (%)	447 (61.4)	32 (50)	0.074
Periodontal diagnosis at baseline and treatment no (%)			0.827
Stage 1,2 untreated:	84 (12.56)	7 (13.46)	
Stage 1,2 treated:	173 (25.86)	11 (21.15)	
Stage 3,4 untreated:	138 (20.63)	13 (25)	
Stage 3,4 treated:	274 (40.96)	21 (40.38)	
Implant type (BEGO vs. MEGAGEN), no (%)	68 (9.34)	8 (12.5)	0.411
Prosthetic, no (%)			0.116
Cemented:	249 (34.2)	26 (40.62)	
OD:	101 (13.87)	13 (20.31)	
Screw:	378 (51.92)	25 (39.06)	
Loading, no (%)			0.373
Conventional:	523 (71.84)	41 (64.06)	
Early:	99 (13.6)	10 (15.62)	
Immediate:	106 (14.56)	13 (20.31)	
No of prosthetic units/implant ≤ 3, no (%)	722 (99.18)	61 (95.31)	*0.03*
Implant position, no (%)			0.424
Mandibular anterior:	49 (6.73)	4 (6.25)	
Mandibular posterior:	270 (37.09)	25 (39.06)	
Maxillary anterior:	110 (15.11)	14 (21.88)	
Maxillary posterior:	299 (41.07)	21 (32.81)	
Bone reconstruction before implant placement, no (%)			0.896
Iliac crest:	9 (1.24)	0 (0)	
GBR:	11 (1.51)	0 (0)	
No:	562 (77.3)	54 (84.38)	
Onlay graft:	39 (5.36)	4 (6.25)	
Alveolar grafting:	38 (5.23)	3 (4.69)	
Buccal reconstruction:	5 (0.69)	0 (0)	
Sinus lift:	63 (8.67)	3 (4.69)	
Bone reconstruction and implant placement, no (%)			0.758
No:	389 (53.43)	32 (50)	
Onlay graft:	0 (0)	0 (0)	
Alveolar grafting:	59 (8.1)	7 (10.94)	
Buccal reconstruction:	226 (31.04)	23 (35.94)	
Sinus lift:	36 (4.95)	2 (3.12)	
Socket shield:	3 (0.41)	0 (0)	
Splitting:	15 (2.06)	0 (0)	
Free gingival graft, no (%)	53 (7.28)	7 (10.94)	0.319
Connective tissue graft, no (%)	67 (9.2)	6 (9.38)	0.964
Healing (Open vs. Close), no (%)	205 (28.16)	23 (35.94)	0.188
Recall at a specialized clinic, no (%)	493 (67.72)	48 (75)	0.23
Periodontal check-up at specialized clinic, no (%)	478 (65.66)	47 (73.44)	0.207

OD, overdenture; GBR, guided bone regeneration.

**Table 9:** Global implant success rate (for all implants).

Implant Success:	Yes (n = 1300)	No (n = 127)	*p*-Value
Gender (F vs. M), nr (%)	768 (59.08)	60 (47.24)	*0.01*
Smoker, nr (%)	347 (26.69)	53 (41.73)	*<0.001*
Number of cigarettes per day ≥ 10, nr (%)	325 (25)	52 (40.94)	*<0.001*
Baseline periodontal diagnostic, nr (%)			*<0.001*
Stage 1:	173 (14.46)	16 (14.41)	
Stage 2:	329 (27.51)	23 (20.72)	
Stage 3:	364 (30.43)	16 (14.41)	
Stage 4:	330 (27.59)	56 (50.45)	
Loading, nr (%)			*<0.001*
Conventional:	1001 (77)	78 (61.42)	
Early:	136 (10.46)	25 (19.69)	
Immediate:	163 (12.54)	24 (18.9)	
Healing (open vs. close), nr (%)	320 (24.62)	52 (40.94)	*<0.001*
Recall at a specialized clinic, no (%)	631 (48.54)	87 (68.5)	*<0.001*
Periodontal check-up at specialized clinic, no (%)	600 (46.15)	83 (65.35)	*<0.001*

**Table 10:** Global implant success rate, presenting one implant per patient.

Implant Success:	Yes (n = 550)	No (n = 65)	*p*-Value
Baseline periodontal diagnostic, nr (%)			*<0.001*
Stage 1:	99 (19.88)	10 (18.18)	
Stage 2:	156 (31.33)	14 (25.45)	
Stage 3:	150 (30.12)	8 (14.55)	
Stage 4:	93 (18.67)	23 (41.82)	
Prosthetic design, no (%)			*0.024*
Cemented:	168 (30.55)	24 (36.92)	
OD:	40 (7.27)	10 (15.38)	
Screw:	342 (62.18)	31 (47.69)	
Healing (open vs. closed), nr (%)	133 (24.18)	23 (35.38)	*0.05*
Recall at a specialized clinic, no (%)	269 (48.91)	43 (66.15)	*0.009*
Periodontal check-up at specialized clinic, no (%)	254 (46.18)	40 (61.54)	*0.019*

OD, overdenture.

**Table 11:** Marginal bone level associated with evaluated variables at follow-up.

**A. Marginal Bone Level Associated with the Periodontal Diagnostic at Baseline**						
Periodontal diagnostic baseline:	Stage 1 (n = 48) (a)	Stage 2 (n = 89) (b)	Stage 3 (n = 83) (c)	Stage 4 (n = 66) (d)	*p* {(a,b)/(a,c)/(a,d)/(b,c)/(b,d)/(c,d)} *	
Marginal bone level (mm), median (IQR)	0.1 (0.1–0.2)	0.1 (0–0.2)	0.1 (0.05–0.3)	0.15 (0–0.3)	0.359 {0.661/1/0.972/0.531/0.398/0.969} *	
**B. Marginal bone level associated with treated periodontitis**						
Treated periodontitis:	Yes (n = 192)		No (n = 129)		Difference (95% CI)	*p*
Marginal bone level (mm), median (IQR)	0.1 (0–0.2)		0.2 (0–0.3)		0.1 (−0.1–0)	*0.029*
**C. Marginal bone level associated with periodontitis status**						
Periodontitis status:	Stage 1,2 not treated (n = 43) (a)	Stage 1,2 treated (n = 94) (b)	Stage 3,4 not treated (n = 51) (c)	Stage 3,4 treated (n = 98) (d)	*p* {(a,b)/(a,c)/(a,d)/(b,c)/(b,d)/(c,d)} *	
Marginal bone level (mm), median (IQR)	0.2 (0–0.3)	0.1 (0–0.2)	0.2 (0.1–0.3)	0.1 (0–0.2)	*0.006* {0.581/0.504/0.848/0.005/0.993/0.035}	
**D. Marginal bone level associated with the Implant type**						
Implant type:	BEGO (n = 27)		MEGAGEN (n = 294)		Difference (95% CI)	*p*
Marginal bone level (mm), median (IQR)	0.4 (0.3–0.5)		0.1 (0–0.2)		0.3 (0.2–0.3)	*<0.001*
**E. Marginal bone level associated with the number of prosthetic units per implant**						
Number of prosthetic units/implant ≤ 3:	Yes (n = 318)		No (n = 3)		Difference (95% CI)	*p*
Marginal bone level (mm), median (IQR)	0.1 (0–0.2)		0.1 (0.05–0.15)		0 (−0.1–0.2)	0.602
**F. Marginal bone level associated with the type of prosthesis**						
Type of prosthetic work	Cemented (n = 85) (a)		Overdenture (n = 27) (b)		Screw-retained (n = 209) (c)	*p* {(a,b)/(a,c)/(b,c)} *
Marginal bone level (mm), median (IQR)	0 (0–0.2)		0.1 (0–0.2)		0.1 (0.1–0.2)	*0.001* {0.868/0.005/0.398}
**G. Marginal bone level associated with implant position**						
Implant position:	Anterior mandible (n = 9) (a)	Posterior mandible (n = 147) (b)	Anterior maxilla (n = 29) (c)	Posterior maxilla (n = 136) (d)	*p* {(a,b)/(a,c)/(a,d)/(b,c)/(b,d)/(c,d)} *	
Marginal bone level (mm), median (IQR)	0.2 (0.2–0.3)	0.1 (0–0.25)	0.2 (0.1–0.3)	0.1 (0–0.2)	*0.042* {0.071/0.747/0.007/0.757/0.49/0.281}	
**H. Marginal bone level associated with healing type**						
Healing:	Open (n = 88)		Closed (n = 233)		Difference (95% CI)	*p*
Marginal bone level (mm), median (IQR)	0.1 (0–0.2)		0.1 (0–0.2)		0 (0–0)	0.611
**I. Marginal bone level associated with recalls at specialized clinics**						
Recall at a specialized clinic:	Yes (n = 219)		No (n = 102)		Difference (95% CI)	*p*
Marginal bone level (mm), median (IQR)	0.1 (0–0.2)		0.2 (0.1–0.3)		0.1 (−0.1–−0.1)	*<0.001*
**J. Marginal bone level associated with periodontal check-ups at specialized clinics**						
Periodontal check-ups at a specialized clinic:	Yes (n = 211)		No (n = 110)		Difference (95% CI)	*p*
Marginal bone level (mm), median (IQR)	0.1 (0–0.2)		0.2 (0.1–0.3)		0.1 (−0.1–−0.1)	*<0.001*
**K. Marginal bone level associated with the prosthetic loading time**						
Prosthetic loading:	Conventional (n = 561) (a)		Early (n = 107) (b)		Immediate (n = 119) (c)	*p* {(a,b)/(a,c)/(b,c)} *
Marginal bone level (mm), median (IQR)	0.1 (0–0.2)		0.1 (0.05–0.3)		0.1 (0–0.2)	*0.017*{0.113/0.193/0.011}

IQR, interquartile range; CI, confidence interval; *, the global test *p*-value for the comparison between all groups, followed by posthoc *p*-value tests comparisons between the groups indicated by letters between the brackets.

**Table 12:** Generalized linear mixed-effects regression model predicting marginal bone level, taking into account the patient correlation.

Characteristic	Estimate	95% CI	*p*-Value
(Intercept)	0.185	(0.112–0.258)	
Gender (M vs. F)	0.018	(−0.009–0.045)	0.186
Osteoporosis	−0.034	(−0.084–0.017)	0.190
Periodontal diagnosis baseline/grouped			*0.0037*
Stage 1,2 vs. healthy, edentulous	0.027	(−0.027–0.081)	
Stage 3,4 vs. healthy, edentulous	0.067	(0.014–0.119)	
Previously treated periodontitis	−0.011	(−0.043–0.021)	0.4975
Implant type MEGAGEN vs. BEGO	−0.201	(−0.241–−0.16)	<0.001
Prosthetic			*0.019*
OD	0.027	(−0.024–0.078)	
Screw-retained	0.035	(0.011–0.06)	
Implant position			0.4195
Posterior mandible	0.018	(−0.02–0.056)	
Anterior maxilla	0.032	(−0.012–0.076)	
Posterior maxilla	0.015	(−0.025–0.056)	
Loading			0.2228
Early	0.038	(−0.005–0.081)	
Immediate	0.006	(−0.031–0.042)	
Recall at a specialized clinic	0.042	(0.013–0.072)	0.005

All the variables are included in the model; OD, overdenture; CI, confidence interval.

## Data Availability

Data that support the findings of this study are not openly available due to the sensitive information that can be used for further research. The data are available from the corresponding author upon reasonable request.
